# Does movement efficiency differentiate performance? An analysis of competition load in elite women’s padel

**DOI:** 10.5114/biolsport.2026.159560

**Published:** 2026-05-20

**Authors:** Ricardo Miralles, José Guzmán, Rafael Martínez-Gallego, Diego Muñoz, Jesús Ramón-Llin

**Affiliations:** 1 Research Group of the Valencian Community Padel Federation; 2 Research Group on Sports Technique and Tactics, University of Valencia, Valencia, Spain; 3 Faculty of Spors Sciences, University of Extremadura, Cáceres, Spain

**Keywords:** Women’s padel, EPTS, Racquet sport, Accelerometery, Sports performance

## Abstract

Quantification of competition load provides critical information on player performance. This study aimed to analyse external loading variables in elite female padel players to establish reference values and distinguish mobility profiles between match-winning and match-losing pairs. Kinematic data were collected from 121 professional players during 34 official matches using Wimu Pro EPTS inertial devices. Descriptive analysis established normative values for elite competition: players covered a mean total distance of 3648 m (Mdn = 3317 m; IQR = 1312.50) and reached a mean maximum velocity of 14.14 km/h (Mdn = 14.17 km/h; IQR = 3.42). The median PlayerLoad was 54.04 arbitrary units (a.u.) (IQR = 23.64). Regarding high-intensity actions, players performed a median of 491 accelerations and 441 decelerations per match. Comparative analysis revealed that losing pairs covered greater total distance (Mdn = 3364 m) compared to winners (Mdn = 3103 m), though this difference was trivial and non-significant (p = 0.719; r = -0.03). Similarly, losers performed more absolute accelerations (Mdn = 525 vs. 467) and relative accelerations per hour (Mdn = 356 vs. 339; p = 0.275; r = -0.10) compared to winners. This research provides objective accelerometry-based reference values for elite women’s padel. The results indicate that locomotor demands are broadly similar between winning and losing pairs. Although greater total distance and acceleration volume were noted in losing players, these differences were small and not statistically significant.

## INTRODUCTION

The increase in the number of padel players and competitions at regional, national and international levels has been extremely important for the expansion of this sport worldwide. Aspects such as easy learning, greater media promotion and television broadcasting and its low-impact nature have contributed significantly to the popularization of this sport [1, 2, 3]. This popularization has led to greater professionalization of the players, and consequently growing research focused on the analysis of performance and aspects related to the game [4, 5]. However, most studies to date have focused on technical-tactical or time-related aspects, with limited evidence on the specific physical demands experienced by elite female players during matches [6, 7]. Understanding these demands is essential for designing training programmes tailored to actual competitive requirements.

Notational analysis, understood as the recording of dynamic and complex actions of the game for subsequent analysis, has evolved to be considered performance analysis with the incorporation of the analysis of physical parameters, providing a comprehensive view of each sport [8]. In high-performance sports, these records are used by technical bodies to evaluate changes in the players’ performance, avoid possible injuries and prevent overloads [9]. The latest studies have classified the analysis of the influential variables in padel into technical-tactical, anthropometric, physical and displacement demands and psychological variables [10, 11]. There is a scarcity of studies that quantify the training loads in padel, attending to the demands of the competition. Training load refers to the quantification of the work performed by athletes, providing objective information that can support decision-making related to fatigue management and performance optimization when appropriately monitored [12]. Quantification of competition demands allows for improved specificity of training loads [11]. The use of GPS devices in padel to quantify the mechanical load produced by stroke volume, stroke intensity, and accelerations (acc+) and decelerations (dec+) could be of great interest. This technology has been used considerably in many team sports such as rugby union, rugby sevens, Australian league soccer, basketball, field hockey, soccer, and cricket, each with their own individualized approach to obtaining the desired data [13, 14, 15] and more recently, in tennis [16] and padel [17]. For this, external loading is used, i.e., quantifying the load required by a game situation, which is applied equally to all players [18, 19].

Studies have characterized the external load in women’s padel in three fundamental aspects: on the one hand, the time structure; the duration of points in women’s padel is superior to men’s padel. In the female category, a match duration of between 0:42:00 and 1:29:15 (h:mm:ss) has been reported, with playing and resting times of 9.67–16.80 s and 20.3–28.8 s, respectively [3]. Torres-Luque et al. [20] observed that the duration of points is usually distributed across 3 to 6 s (23.2%), 6 to 9 s (29.3%) and 9 to 12 s (19.6%) in both sexes [20]. Female matches present a higher duration of points, strokes per point, actual playing time, rest time, rest time per point and points per match compared to male matches [21, 22]. Regarding the work-to-rest ratio, similar values were found between both sexes [21]. Therefore, female players constantly try to adopt technical-tactical behaviours and actions, which define different styles of play [23]. These styles of play will possibly mark the movements that occur on the court.

Thus, we can define padel as an intermittent sport, which alternates periods of high intensity work, in which the player must be able to perform movements with continuous changes of direction and execute different technical actions with periods of rest and recovery between efforts [24]. The profile of the physical demands of athletes is determined by the characteristics of the actions that they must perform in competition to solve the motor task [25]. It will be essential to know what these characteristics are in order to be able to propose training according to and adapted to the padel players that will allow them to respond in the best possible way to the physical demands they face on a padel court.

Therefore, electronic performance tracking systems (EPTS) have emerged as one of the most important components of monitoring. These systems consist of various sensors, including accelerometers, gyroscopes, and magnetometers [17, 26, 27, 28], which allow for the assessment of external load based on the distance covered. In addition, they capture additional components of acceleration that occur during jumps and turns, addressing certain limitations of previous studies [29]. Thus, accelerometery-derived measures of workload during match play vary based on manufacturer-specific processing systems (e.g., PlayerLoad), although they report the sum of acc+ from running, jumping/landing, and rotations in a.u. [16, 30].

In this context, it becomes necessary to accurately describe the external match demands in elite women’s padel. By identifying key physical load variables, such as distance covered, acc+, dec+, and PlayerLoad, coaches and practitioners can better adapt training strategies to reflect the realities of match play [31, 32]. Despite the growing popularity of the sport, there is still a lack of detailed data in this area, particularly for female athletes.

To date, no studies have reported values for acc+, dec+, highspeed running (HSR), total and explosive distance, or PlayerLoad in elite female padel. Therefore, information on load quantification in female padel players could be fundamental.

Thus, the objective of this research was to assess external competition demands in elite women’s padel. Specifically, the study analysed potential disparities in physical performance metrics according to match outcome (winning vs. losing pairs).

## MATERIALS AND METHODS

### Sample

The sample consisted of 136 player-match observations collected from 34 official matches (12 finals and 22 semi-finals) of the World Padel Tour during the 2021–2022 season. The participants were 121 unique female professional players aged between 18 and 34 years, with a World Padel Tour ranking between 30 and 403. All matches were official, and players participated voluntarily, signing an informed consent form prior to the study.

Data collection covered the entire annual competitive calendar. This longitudinal approach captures performance across all phases of the season. All matches were played outdoors in the Valencian Community (Spain). While specific environmental metrics were not recorded for each match, the region features a Mediterranean climate with average historical temperatures ranging from 12°C to 26°C and a relative humidity of approximately 60–70% during these months.

### Instrument

The WIMU PRO device (RealTrack Systems, Almeria, Spain) was used. This device has been shown to have validity in positioning and tracking in previous studies [33, 34]. It integrates multiple sensors: four 3D accelerometers (± 16 g, ± 32 g, and ± 400 g) sampling at 100 Hz, three 3D gyroscopes (2000°/s) at 100 Hz, and a GNSS chip sampling at 18 Hz. To ensure data validity for high-intensity movements, the system employs Sensor Fusion technology, combining inertial data with positional data. Specifically, accelerations, decelerations, and PlayerLoad metrics are derived from the high-frequency inertial sensors (100 Hz), ensuring precision during the short, explosive movements typical of padel, while distance and velocity are tracked via the 18 Hz GNSS unit.

### Procedure

The data analysed in this study were obtained from the official monitoring records of the Valencian Community Padel Federation. In each match, data were taken from each player, with the informed and signed consent of the players. Before the general warm-ups, the players were fitted with the vest without the Wimu Pro device. Once they had performed their specific warm-up exercises and just 3 minutes before the start of the test, the Wimu Pro device (RealTrack Systems, Almería, Spain) was placed in the vest, turned on, and data recording began when the player hit the first serve of the match. Data recording was completed when the last point of the match ended. The study was conducted in accordance with the principles of the Declaration of Helsinki.

### Variables

Player load is the accumulation of effort that an athlete has endured during a given activity. Measures of player load can be categorized as internal or external [35, 36, 37].

The variables analysed are described in Table 1; in the case of the dependent variables, they have been grouped according to criteria of load volume and intensity [17, 31]:

**TABLE 1:** Description and definitions of the external load variables analysed.

Group of variables	Variables	Definition
	Duration (s)	Total duration of the match.
	Distance (m)	Total Distance covered during the match.
	Distance_Rel (m)	Total Distance covered, normalized per hour of play.

**Volume and intensity classical variables**		
	Max_Speed_(km/h)	Highest Speed reached by the player during the match.
	Accelerations	Total number of accelerations exceeding +1.12 m/s^2^
	Accelerations_Rel	Total number of decelerations exceeding -1.12 m/s^2^
	Max_Acceleration (m/s^2^)	Total number of accelerations ≥ 1.12 m/s^2^, normalized per hour of match.
	Decelerations	Maximum acceleration achieved.
	Decelerations_Rel	Maximum deceleration achieved.
	Max_Deceleration (m/s^2^)	Total number of decelerations ≤ -1.12 m/s^2^, normalized per hour of match

**Volume and intensity novel variables**		

	Player_Load_(a.u.)	Vectorial sum of accelerations in the three axes (vertical, anteroposterior, and lateral). Used to assess total neuromuscular load. $d=\frac{\sqrt{{\left(a_{y1}-a_{y-1}\right)}^{2}+{\left(a_{x1}-a_{x-1}\right)}^{2}+{\left(a_{z1}-a_{z-1}\right)}^{2}}}{100}$
	Explosive_Distance_(m)	Total distance covered with accelerations > 1.12 m/s^2^
	Explosive_Distance_Rel (m)	Distance covered with accelerations > 1.12 m/s^2^, normalized per hour of session.
	HSR_Rel_(m)	Relative High-Speed Running: Distance covered at speeds above 75.5% of the player’s historical maximum speed.

Note: a.u. = arbitrary units; HSR = High Speed Running; Rel = Relative to 1 hour of play; Max = Maximum.

### Acceleration and deceleration profile calculation

Accelerations and decelerations were identified using a threshold of ≥ 1.12 m · s^−2^. Although sensitivity to different thresholds exists, this specific cut-off was selected to align with the manufacturer’s default processing algorithms (SPRO Software, RealTrack Systems) and, most importantly, to ensure direct comparability with the emerging corpus of elite padel literature [17, 24, 31]. This consistency allows for the establishment of robust reference values across professional competitions.

To determine the distance covered during high-intensity actions, a specific event-detection algorithm was applied using SPRO software. An acceleration or deceleration event was defined as a continuous period where the inertial sensor output exceeded the threshold of ± 1.12 m · s^−2^.

Onset: The specific timestamp when acceleration increased above 1.12 m · s^−2^.

Offset: The timestamp when acceleration dropped below the threshold.

Distance per event: Calculated as the trajectory accumulated between the onset and offset times.

### High-speed running (HSR)

Distance covered at speeds above 75.5% of the player’s historical maximum speed. This individual reference value was determined as the peak velocity recorded for each player across the entire 2021–2022 season (multi-match analysis) to avoid underestimation derived from single-match or session-specific maximums. The 75.5% threshold (manufacturer default, SPRO) was selected to provide an individualized intensity metric suitable for the short-court dimensions of padel, where traditional absolute high-speed bands are less sensitive.

### Data analysis

For data analysis, IBM SPSS Statistics (version 28.0; IBM Corp., Armonk, NY, USA) and RStudio (RStudio Team, 2022) were used to create the graphs. The median and interquartile range were used for descriptive purposes. Initially, Kolmogorov–Smirnov tests for normality were performed. Wilcoxon tests were performed to compare load variables as a function of match outcome (winners vs. losers). Effect size was calculated from Pearson’s r and classified as follow: 0.5 – large effect, 0.3 – medium effect, and 0.1 – small effect. Significance was adjusted for p values < 0.05.

## RESULTS

### Descriptive analysis

Table 2 shows the descriptive values of the competitive load variables analysed. The players covered a mean distance of 3648 m per match, with a median of 3316 m (IQR = 1312.50). The relative distance covered per hour was 2342 m, with a median of 2148 m (IQR = 496). As for the maximum speed reached, the players registered a mean speed of 14.14 km/h, with a median of 14.17 km/h (IQR = 3.42). The PlayerLoad showed a median of 54.04 a.u. (IQR = 23.64). Regarding high-intensity actions, players performed a median of 491 acc+ (IQR = 230) and 441 dec+ (IQR = 213).

**TABLE 2:** Descriptive statistics of competition load variables in elite women’s padel.

Variable	Mean	Median (IQR)
**Volume variables**		
Duration (s)	5534	5005 (2310)
Distance (m)	3648	3317 (1313)
Distance_Rel (m)	2342	2148 (495)

**Volume and intensity classical variables**		
Max_Speed_(km/h)	14.14	14.17 (3.42)
Accelerations+1	524	491 (230)
Accelerations_Rel	351	352 (113)
Max_Acceleration (m/s^2^)	4.25	4.13 (0.62)
Decelerations+1	450	441 (213)
Decelerations_Rel	295	313 (104)
Max_Deceleration (m/s^2^)	-4.30	-4.14 (0.84)

**Volume and intensity novel variables**		
Player_Load_(a.u.)	57.07	54.04 (23.64)
Explosive_Distance_(m)	305	298 (170)
Explosive_Distance_Rel (m)	201	201 (93)
HSR_Rel_(m)	10.96	6.53 (7.02)

Note: IQR = interquartile range; a.u. = arbitrary units; HSR = High Speed Running; Rel = Relative to 1 hour of play; Max = Maximum; Player Load = the vector sum of accelerations on the three axes; Explosive Distance: refers to the total distance covered with an acceleration greater than 1.12 m/s^2^.

### Analysis according to the match result

Table 3 shows the movement variables as a function of match outcome. Losers covered a longer distance (Mdn = 3364, IQR = 1152) compared to winners (Mdn = 3103 m, IQR = 1486), although this difference was not significant (p = 0.719; r = -0.03). Similarly, no significant difference was found in the relative distance covered per hour (p = 0.730; r = -0.03).

**TABLE 3:** Analysis of competition load variables based on match outcome.

Variable	Winner Mdn (IQR)	Loser Mdn (IQR)	W	p	r
**Volume variables**					
Duration (s)	5005 (2310)	5005 (2350.75)	1816.50	0.946	0.01
Distance (m)	3103 (1486)	3364 (1152)	1760	0.719	0.03
Distance_Rel (m)	2148 (492)	2152 (489)	1763	0.730	0.03

**Volume and intensity classical variables**					
Max_Speed_(km/h)	14.01 (3.55)	14.30 (3.18)	1616	0.27	0.10
Accelerations+1	467 (204)	525 (236)	1640	0.33	0.09
Accelerations_Rel	340 (99)	356 (117)	1619	0.28	0.10
Max_Acceleration (m/s^2^)	4.13 (0.68)	4.12 (0.61)	1892	0.75	0.03
Decelerations+1	435 (206)	443 (214)	1730	0.61	0.05
Decelerations_Rel	312 (78)	315 (123)	1737	0.63	0.04
Max_Deceleration (m/s^2^)	-4.28 (0.90)	-4.07 (0.81)	1594	0.22	0.11

**Volume and intensity novel variables**					
Player_Load_(a.u.)	49.53 (28.76)	58.98 (19.50)	1532	0.12	0.14
Explosive_Distance_(m)	283 (167)	308 (168)	1724	0.58	0.05
Explosive_Distance_Rel (m).	198 (72)	202 (113)	1752	0.69	0.13
HSR_Rel_(m)	6.89 (6.31)	6.51 (7.15)	1681.50	0.44	0.07

Note: Mdn = median; IQR = interquartile range; a.u. = arbitrary units; HSR = High Speed Running; Rel = Relative to 1 hour of play; Max = Maximum; Player Load = the vector sum of accelerations on the three axes; Explosive Distance: refers to the total distance covered with an acceleration greater than 1.12 m/s^2^.

As for the acc+ and dec+ variables, the losers recorded a greater number of acc+, with a median of 525 acc+ (IQR = 236), while the winners recorded a median of 467 acc+ (IQR = 204). Relative acc+ were also higher in the losers, with a median of 356 relative acc+ (IQR = 117), versus 339 relative acc+ (IQR = 99.05) in the winners (p = 0.275; r = -0.10).

As for dec+, the losers also presented higher values, with a median of 443 dec+ (IQR = 214) compared to 435 Dec+ (IQR = 206 in the winners (p = 0.606; r = -0.05). Looking at relative dec+, the losers recorded a median of 315 relative dec+ (IQR = 123), while the winners recorded 312 relative dec+ (IQR = 78) (p = 0.632; r = -0.04).

### Acceleration and deceleration profile

**FIG. 1:**
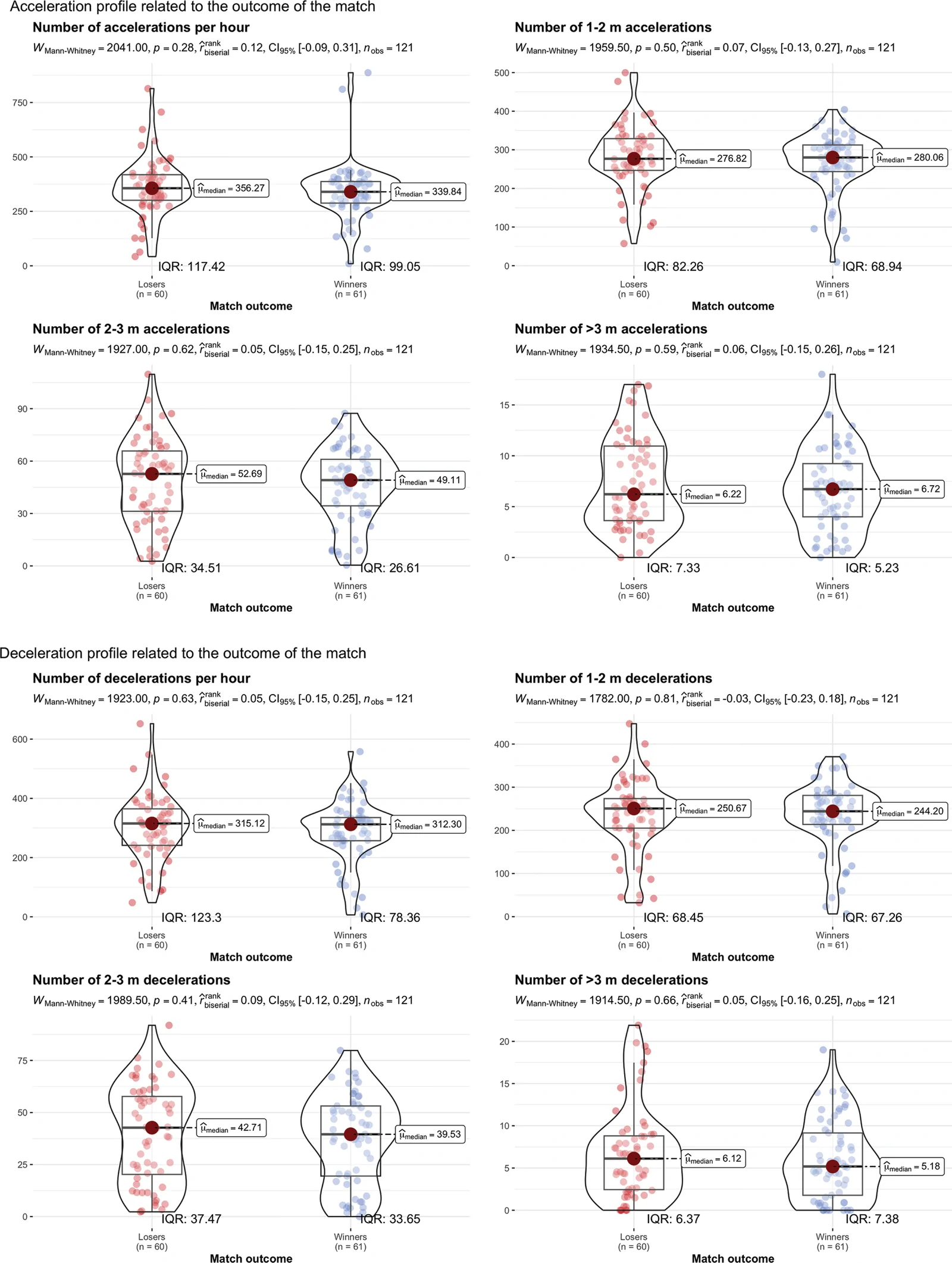
Acceleration and deceleration profile depending on match outcome.

Figure 1 shows the profile of acc+ and dec+ as a function of match outcome. Regardless of the match outcome, about 80% of acc+ occurred at distances of 1–2 m. Regarding the differences as a function of the outcome, although no significant differences were found between winners and losers, the losers performed a higher number of acc+ and dec+ at all distances, except for acc+ greater than 3 m. In particular, in the 1 to 2 m range, losers performed a median of 277 acc+, while winners performed 280.06 acc+ (p = 0.50; r = 0.07). Similarly, over the same range of distances, losers performed a median of 256 dec+, while winners performed 284 dec+ (p = 0.07; r = 0.23).

## DISCUSSION

This study presents the first accelerometry-based quantification of competition load in elite female padel. The results define a highintensity, intermittent profile, where approximately 80% of accelerations occur over short distances (1–2 m) and players cover an average of ~3648 m per match. Although no significant differences were found based on match outcome, losing pairs exhibited a higher total distance and acceleration volume compared to winners. These findings fill a gap in the literature by providing novel reference values for professional female players using EPTS technology.

### Volume load variables

In relation to the distance covered, the players in our study covered approximately 3317 m, while the distance per hour of the match was 2148 m. Due to the absence of previous specific data on elite women’s padel, direct comparisons with female peers are not yet possible. However, when contrasting our findings with elite male players, Miralles et al. [17] reported slightly higher values for total distance (3430 m) and relative distance (2401 m/h). In this study, the disputed matches had a duration of 83 min (median), which was lower than the results reported by Torres-Luque et al. [20] in professional female players. In addition, other authors observed distances between 2310 m and 3440 m in different levels of male amateur play [38]. The evolution of padel can be reflected in the volume of movements, due to the different styles of play and the constant improvement of the technical-tactical actions of the players (01). In addition, as reported in recent works [39], the number of errors committed by the players was reduced in recent seasons, and even these same authors made a prediction of a further decrease in errors in the coming years. This could lead to an increase in the volume of movement of the players. These results provide relevant information for coaches and physical trainers to make the training of players more specific, taking into account the distances covered, where it seems that a greater volume of effort implies greater resistance work.

### Classic volume and intensity load variables

The maximum speed of displacement that the female players registered during the matches played was 14.14 km/h (median values), with no similar studies in this population to date. Miralles et al. [17] report maximum speed of 15.21 km/h in elite men’s padel. Also, Ramón-Llin et al. [29] reported results with elite padel players, who reached speeds of 2.5 km/h, well below that demonstrated in this study, with different methods of determining speeds. Amieba and Salinero [40] reported average speed of 2.59 km/h, also adding that 80% of the movements were performed at speeds of less than 6 km/h, although in the amateur category. However, these authors observed a maximum speed of 15.4 km/h, similar to that observed in our study. It is crucial to note that while median relative distances (~2148 m/h) reflect a moderate overall pace, these average metrics may underestimate the true neuromuscular intensity of the game. The presence of maximum accelerations (> 4 m/s^2^) and decelerations (> -4 m/s^2^) highlights that elite female padel involves critical highintensity spikes. These peak values represent the “most demanding scenarios” in terms of neuromuscular load, requiring players to repeatedly generate maximal force in short timeframes, a demand that average values often mask. Also, Castillo-Rodriguez et al. [41] observed average speed of 2.09 km/h in male players of different playing categories, with a very low percentage of high-speed actions (13.6–18 km/h). These lower speed data could be related to the greater number of strokes made from the middle zone [20, 22] or the back of the court, using the technical action of the tray and the lob [21]. In addition, as observed by Martin et al. [42], the intensity of play in women’s padel is lower than in men’s padel, so the speeds achieved may also be lower. Thus, as reported by other authors [38], maximum speeds are usually reached when the player sprints to the net, and the female players would likely be located in that middle zone.

On the other hand, in our results, maximum dec+ of -4.14 m/s^2^ and maximum acc+ of 4.13 m/s^2^ were recorded. To date, this is the first study to analyse these parameters in women’s padel. Previous studies carried out in male players [17] obtained maximum dec+ of -4.52 m/s^2^ and maximum acc+ of 4.60 m/s^2^. These results support the idea that sprints to the net and exiting the court during a smash are more characteristic of men’s padel. Recently, Martín-Miguel et al. [7] observed a higher intensity of play in male padel, compared to female padel, with game rates of 0.8 strokes per second and 0.73 strokes per second, respectively. This higher intensity could explain the higher acc+ and dec+ of male players.

When contextualizing these demands within the spectrum of racket sports, comparisons with tennis provide crucial insight into the unique physiological signature of padel. While competitive tennis players typically cover greater total distances per match due to larger court dimensions [25], our data reveal that padel places a higher emphasis on movement density rather than volume. Unlike the movement profiles observed in tennis, which often involve longer displacement phases [16], padel involves a predominance of shortdistance efforts (< 2 m). This suggests that the intensity of padel is driven by the high frequency of accelerations and decelerations (neuromuscular load) rather than the total volume of linear running, highlighting the importance of training reactive agility over the aerobic capacity typically prioritized in tennis.

### Advanced external load indicators

The players in our study recorded mean values of 54.04 a.u. in PlayerLoad. Recently, in men’s padel they have also observed similar values to these, with a PlayerLoad of 53.88 for right-sided players and 56.41 for left-sided players [31]. On the other hand, studies in female tennis have reported movement profiles and stroke demands that suggest a high neuromuscular load, comparable to our findings in padel [25]. Furthermore, different load responses have been recorded across matches, suggesting that coaches should be able to monitor match loads to be able to reproduce them in training in order to optimize the training load prescription according to the demand of each match [42].

### Effect of match outcome

Analysis based on match outcome revealed no statistically significant differences in external load variables, indicating that physical demands are largely homogeneous across elite competitors regardless of the match result. However, losing pairs covered greater distances and performed higher frequencies of acc+ and dec+ compared to winners. About 80% of acc+ occurred at distances of 1–2 m. The losers performed a greater number of acc+ and dec+ at almost all distances. A recent study in junior male tennis players found that winners had lower movement player load than losers, as we observed in our study, without reaching statistical significance [16]. In padel, winning pairs spend more time at the net, producing more winners and generators of forced error through overhead shots and volleys, while losing players commit more forced and unforced errors through any type of shot [42]. It is possible that the losing players, spending more time in the backcourt zone, have to make a greater number of displacements and, therefore, more acc+ and dec+ than the net players. This factor can be very important when planning training sessions at the net and at the back court, where the external load demands can be different. In addition, this higher number of acc+ and dec+ could lead to a higher number of errors in the losing pairs [43].

### Limitations

This study has several limitations. First, only external load variables were analysed, and no measures of internal load (heart rate, perceived exertion, or biochemical markers) were included, which would have provided a more comprehensive understanding of players’ physiological responses. Second, although the use of EPTS technology (Wimu Pro) allowed precise quantification of kinematic demands, contextual and tactical factors—such as court positioning, shot selection, or rally characteristics—were nor examined, which limits the interpretation of load profiles in relation to playing style. Third, the sample was composed exclusively of elite professional female players, so the results cannot be generalized to amateur or youth populations. Finally, current literature has extensively documented side-specific demands and match loads in male players [17, 29]. However, future research should urgently replicate these analyses in the female category to determine whether the asymmetries observed in men (e.g., higher workload on the left side) are also present in elite women. Additionally, longitudinal monitoring of these variables throughout a full season is necessary to understand load fluctuations and optimize periodization. Future studies should also aim to integrate internal load monitoring (heart rate, RPE) with these kinematic variables to provide a comprehensive understanding of the physiological cost of competition in women’s padel.

### Practical implications

The results of this study provide valuable insights for coaches, physical trainers, and performance analysts working with elite female padel players. Firstly, the quantification of external load variables—such as total and relative distance covered, acc+ and dec+ profiles, and PlayerLoad—allows practitioners to better tailor training sessions to match the physical demands observed in real competition.

Specifically, losing pairs exhibited a higher number of acc+ and dec+ and covered greater distances; however, these differences were not statistically significant, and may have been due to increased time in defensive positions. If supported by further research, such findings may have practical implications for training, suggesting the need to prepare athletes for high neuromuscular loads and repeated shortdistance efforts, particularly in backcourt scenarios. Accordingly, coaches may consider incorporating drills that simulate these specific movement demands and emphasize recovery strategies to prevent fatigue-related errors.

Furthermore, understanding the acc+ and dec+ profiles, particularly the predominance of actions within the 1–2 m range, can inform the design of position-specific conditioning programmes. Differentiating training loads according to match scenarios and player roles (e.g., net play vs. baseline play) could enhance performance outcomes and reduce injury risk.

Finally, the use of inertial devices such as EPTS in competition settings proves to be an informative tool for monitoring load, enabling practitioners to bridge the gap between training and match demands more effectively. This evidence-based approach supports individualized training prescriptions that are aligned with the physiological and mechanical realities of elite-level padel.

## CONCLUSIONS

Quantitative analysis indicated that losing pairs performed approximately 12.4% more accelerations (Mdn = 525) than winning pairs (Mdn = 467); however, this difference was not statistically significant.

Overall differences in total external load were small. Among the analysed variables, decelerations performed over very short distances (1–2 m) demonstrated the largest effect size (r = 0.23); however, this difference was not statistically significant and should be interpreted with caution. This pattern may reflect a higher frequency of braking actions associated with defensive adjustments and positional corrections during play.

Although the present findings do not support definitive conclusions regarding performance determinants, they highlight short-distance acceleration–deceleration actions as a component warranting further investigation.

From an applied perspective, these results tentatively suggest that training interventions for elite female players could benefit from incorporating drills that emphasize repeated short-distance accelerations and decelerations under game-like constraints. Such drills may help replicate scenarios characterized by high defensive density and frequent micro-adjustments, while acknowledging that further research is required to confirm their relationship with match success.
